# The relationship between negative life events and non-suicidal self-injury (NSSI) among Chinese junior high school students: the mediating role of emotions

**DOI:** 10.1186/s12991-022-00423-0

**Published:** 2022-11-19

**Authors:** Zhongliang Jiang, Zhiyi Wang, Qidi Diao, Jie Chen, Geng Tian, Xiaojing Cheng, Miao Zhao, Long He, Qiang He, Jin Sun, Jintong Liu

**Affiliations:** 1 Department of Child and Adolescent Psychiatry, Shandong Mental Health Center, Shandong University, Shandong, China; 2 Department of Developmental Pediatrics and Child Health Care, The First Affiliated Hospital of Shandong First Medical University and Shandong Provincial Qianfoshan Hospital, Shandong, China

**Keywords:** Life events, Non-suicidal self-injury, Emotion, Chinese junior high school students, Prevalence

## Abstract

**Background:**

Adolescent non-suicidal self-injury (NSSI) is common and adolescence is the most common period of first self-injury, and the occurrence of NSSI is influenced by negative life events and emotional symptoms. The mediating role of emotional symptoms in the interaction between negative life events and NSSI has not been carefully investigated yet.

**Methods:**

For middle school students in three schools in a Chinese province, the Adolescents Self-Harm Scale was used to investigate NSSI, the Adolescent Self-Rating Life Events Check List was used to investigate adolescent negative life events, and the Self-Rating Anxiety Scale and Self-Rating Depression Scale were used to assess their emotional symptoms. After the description of general data and the test for differences between groups, the relationship between negative life events, emotional symptoms and NSSI was analyzed using Pearson correlation analysis. Structural equation modeling was used to analyze the mediating role of emotions in negative life events and NSSI.

**Results:**

A total of 2376 junior high school students completed this survey, which revealed an annual NSSI prevalence of 37.1% (*n* = 881) and a higher prevalence of NSSI among girls and rural adolescents. Among adolescents who developed NSSI, 67.4% (*N* = 594) used multiple means of self-injury. The most common means of self-injury was hair pulling (51.0%), and the most common NSSI purpose and external factors/events were venting bad emotions or feelings (57.5%) and poor academic performance (44.9%), respectively. Negative life events, emotional symptoms and NSSI were positively associated (*P* < 0.05). Structural equation modeling with negative life events, emotional symptoms and NSSI as variables showed that the model-fit index matched the data well, with RMSEA = 0.073, AGFI = 0.945, GFI = 0.980, CFI = 0.985, NFI = 0.982, TLI = 0.968, IFI = 0.985, and negative life events, emotional symptoms (anxiety, depression) and NSSI all had direct effects with standardized path coefficients of 0.16, 0.19, and 0.23, respectively, with negative life events playing an indirect role in NSSI through emotional symptoms and emotional symptoms playing an incomplete mediating role in negative life events and NSSI.

**Conclusion:**

The prevalence of NSSI was higher among Chinese junior high school students. Both negative life events and emotional symptoms were direct risk factors for NSSI. In addition, negative life events were also indirect risk factors for NSSI, and emotional symptoms played an incomplete mediating role in the relationship between the effects of negative life events and NSSI. This indicates that the combination of reducing the frequency of negative life events while maintaining individual emotional stability during adolescent development can effectively reduce the prevalence of NSSI in adolescents.

## Introduction

Non-suicidal self-injury (NSSI) is a direct and deliberate act of hurting oneself without the purpose of suicide, including various forms such as cutting, burning, and biting oneself [1]. Adolescent NSSI has become a behavioral problem that we must pay attention to, and the Diagnostic and Statistical Manual of Mental Disorders Fifth edition (DSM-5) states that NSSI most commonly begins in early adolescence and persists for many years, most often with the goal of reducing negative emotions. Carr et al. [2] suggested that stimuli generated before the onset of the NSSI and the consequences after the onset of the NSSI would have a reinforcing effect on the NSSI, causing individuals to act similarly when presented with similar situations again. On this basis, Nock et al. proposed a “four-function model” of NSSI, outlining four main functions of NSSI, namely: automatic-positive reinforcement (e.g., experiencing stimuli or pain), automatic-negative reinforcement (e.g., reducing negative emotions), social-positive reinforcement (e.g., gaining others' attention), Social-negative reinforcement (e.g., avoidance of interpersonal task demands) [3]. Nock et al. also state that self-injury peaks in adolescence [4] and is the most common period when self-injury first occurs [5]. Factors influencing NSSI include individual factors, social-environmental factors and family factors [6]. Common risk factors such as emotional sensitivity and vulnerability [7], having suffered abuse in the family [8], and poor relationships with peers [9]. In the short term, the damage caused by NSSI to adolescents is mainly physical damage. But in the long term, NSSI can also cause significant psychological damage to adolescents, such as knife wounds and scars. The marks of self-injury may receive strange looks from classmates, and may be regarded as “problem child” by others. One study found [10, 11] that individuals with NSSI cognitively distinguished between NSSI and suicidal behavior. However, NSSI often precedes suicide [12] and NSSI is a significant predictor of suicide [13]. Previous studies have shown that among patients with a history of NSSI, 70% have attempted at least one suicide and 55% have attempted multiple suicides [14]. Therefore, NSSI must draw our attention.

Negative life events may cause changes in stress state and promote a series of physiological and psychological changes [15]. Adolescence is an important period of psychological development and change in a person's life. Adolescents in this period may face a series of negative events from family, school, physical and psychological aspects, such as family financial difficulties, tension with teachers, discrimination and ostracism by classmates, failure in exams, etc. These negative life events do not exist in isolation; for example, adolescents from economically struggling families may receive discrimination and ostracism from others at school; tension with the teacher of a course may lead to dislike of the course and consequent failure in the exam.

Negative life events can produce physically and psychologically distressing experiences in individuals, which in turn can lead to adverse emotions in adolescents. Adolescents who are subjected to severe levels of negative life events or who are less able to withstand setbacks may develop anxiety and depression. Previous studies have shown that negative life events are one of the psychosocial factors that induce emotional problems in adolescents, especially in inducing anxiety [16]. Penner-Goeke et al. showed an increased risk of depression after exposure to negative life events. [17]. A previous survey of Chinese adolescents showed that depression can act as a mediator between stressful life events and the occurrence of NSSI [18]. In addition, other studies have also shown that anxiety and depression are risk factors for NSSI [19–21]. Another study [13] showed that co-morbid anxiety disorders are important risk factors for the development of NSSI behaviors in patients with depressive disorders, and their NSSI behaviors occur more frequently, and the risk of NSSI behaviors in depressive disorders with co-morbid anxiety disorders is three times higher than in those without co-morbid anxiety disorders. Many studies [22–25] around the world have also confirmed that anxiety, depression and NSSI are closely related. Finally, regarding the interrelationship between negative life events and NSSI, previous studies [26–29] have shown that negative life events are a risk factor for NSSI.

Therefore, there are strong associations between negative life events and emotional symptoms, and between symptoms and NSSI. Exploring the interactions between negative life events, anxiety, depression and NSSI and clarifying whether emotional symptoms play a mediating role can help us to intervene effectively in NSSI. This study focused on the mediating role of emotional symptoms in the effect of negative life events on NSSI, using anxiety and depression as observed variables of emotional symptoms.

## Method

### Participants and procedure

We used convenience sampling to select three county-level junior high schools in China (one in the urban area of the county and two in the rural area, with a ratio of about 3:4 students between the two), and a whole-group questionnaire was administered to all students enrolled in the school, with the respondents' grades distributed from the first to the third year of middle school. Prior to the survey, the purpose and significance of the study were introduced to the surveyed students, and written informed consent was obtained from the students and their guardians. To ensure the quality of the survey, classroom teachers were trained accordingly before conducting the survey, including privacy protection, survey process, and discipline maintenance. Classroom teachers were responsible for distributing and retrieving the questionnaires during the survey. The questionnaires were then sorted to eliminate omissions and logical review of unreasonable questionnaires. For adolescents who screen positive for mental illness, classroom teachers will notify the student's guardians and advise them to go to the hospital for medical treatment. In this study, all guardians of the students in the surveyed schools agreed to participate in this survey. A total of 2900 questionnaires were distributed, and after the questionnaires were collated, there were 2376 valid questionnaires, with an effective rate of 81.9%.

## Measures

### General questionnaire

A self-designed general information questionnaire was used to collect personal information from participants, including gender (1 male, 2 female), age, grade, and upbringing (1 urban, 2 rural).

### Adolescents Self-Harm Scale

The Adolescents Self-Harm Scale, developed by Zheng et al. [30] and revised by Feng et al., has good reliability and validity and is widely used in China [31]. The scale lists 15 NSSI modalities and one open-ended NSSI modality fill-in question, which investigates the number of NSSI occurrences in the past year, the degree of harm to the body, the prompting reason or purpose, and the external stimulus event, etc. There are four options for the number of NSSI occurrences, corresponding to scores of 0, 1, 2 and 3. There were 5 options for the degree of physical injury, corresponding to scores of 0, 1, 2, 3 and 4, respectively. The final measure of the severity of the NSSI is the sum of the product of the frequency of each self-injury and the degree of injury, with higher scores considered to be the more severe the subject's NSSI. Cronbach's alpha for this questionnaire = 0.88.

### Adolescent Self-Rating Life Events Check List (ASLEC) [32]

The Adolescent Self-Rating Life Events Check List was developed by Liu et al. and is widely used in China to assess the frequency and intensity of stressful life events among the participants. There are 27 items on the scale, 26 of which are negative life events that may cause psychological reactions in adolescents, and the last item is a self-administered questionnaire for negative life events that are not listed in the questionnaire but have an impact on the participant. The scale is used to determine whether the event occurred within a limited period of time, and if so, the psychological experience at the time of the event is rated on a 5-point scale, i.e., no effect, mild, moderate, severe and very severe, on a scale of 1–5, respectively. If the event did not occur, the scale is based on no effect. The scale can be analyzed on six factors: interpersonal, academic stress, punishment, loss, health adjustment and other, Cronbach's alpha of each factor are 0.85, 0.80, 0.89, 0.79, 0.53, 0.74. In this study, the interpersonal relationship factor, academic stress factor, punishment factor, loss factor and other factor had good reliability coefficients and were included in this study. Cronbach's α = 0.95 for this questionnaire.

### Self-Rating Anxiety Scale (SAS) [33]

The Self-Rating Anxiety Scale is a self-rating scale used to assess the anxiety level of the participants. There are 20 items on the scale and participants rate the frequency of symptoms defined by each item on a 4-point scale, i.e., no or little time, little time, quite a lot of time, most or all of the time. Among them, items 5, 9, 13, 17, 19 are reverse scoring, and the rest items are positive scoring, and the scores of each item are added up to the crude score, and the standard score is obtained by multiplying the crude score by 1.25 and rounding up to integers. Those with a standard score ≥ 50 were assessed as having anxiety symptoms, with higher scores associated with greater anxiety. The questionnaire has been shown to have good reliability and validity, with Cronbach's α = 0.83 [34].

### Self-Rating Depression Scale (SDS) [35]

The Self-Rating Depression Scale is a self-rating scale used to assess the degree of depression of the participant. There are 20 items and participants rate each item on a 4-point scale according to their situation: never or occasionally, sometimes, often and always. Items 2, 5, 6, 11, 12, 14, 16, 17, 18 and 20 are reverse scoring. The scores for each item were summed to a crude score, which was multiplied by 1.25 and rounded to the nearest integers to give a standard score. Those with a standard score ≥ 53 were assessed as having depressive symptoms, with higher scores being associated with greater depression. Cronbach's alpha for this scale = 0.80 [34].

### Statistical methods

Data were analyzed using SPSS 26.0, with measures described by composition ratio [n (%)] or mean ± standard deviation, and differences between groups were compared using independent samples t-tests and rates were compared using χ2 tests. Pearson correlation analysis was used to correlate life events, emotional symptoms and NSSI. The relationship between life events, emotional symptoms and NSSI was modeled using AMOS 24.0. Using RMSEA (Root Mean Square Error of Approximation), AGFI (Adjusted Goodness-of-fit Index), GFI (Goodness-of-fit Index), CFI (Comparative Fit Index), NFI (Normed Fit Index), TLI (Tucker–Lewis Index), IFI (Incremental Fit Index), and SRMR (Standardized Root Mean Square Residual) to evaluate the fit of the model. The values of RMSEA should be lower than 0.08 [36], the values of AGFI, GFI, CFI, NFI, TLI, IFI should be higher than 0.9 [37–41], and the values of SRMR should be lower than 0.08 [42].

## Results

### General

Of the 2900 questionnaires distributed, a total of 2376 junior high school students completed this survey, with a response rate of 81.9%. Among them, 47.8% (*n* = 1136) were male and 52.2% (*n* = 1240) were female, with a mean age of 13.66 ± 0.982. The prevalence of NSSI was 37.1% (*n* = 881). Concerning gender differences, NSSI was reported by 40.2% (499/1240) of females and 33.6% (382/1136) of males, respectively. The difference was statistically significant (*P* < 0.01). The prevalence of NSSI was 28.3% (81/286) in urban junior high school students and 38.3% (800/2090) in rural junior high school students, with a statistically significant difference (*P* < 0.01). In addition, the difference in the prevalence of NSSI by grade level was also statistically significant (*P* < 0.01; Table 1).

**Table 1:** General profile of adolescents in the group with and without NSSI

Item	With NSSI	Without NSSI	*χ*^2^/*t*	*P*
Gender [*n* (%)]			11.120	0.001
Male	382 (43.4)	754 (50.4)		
Female	499 (56.6)	741 (49.6)		
Age ($\overline{x}$ ± *s*)	13.61 ± 1.017	13.69 ± 0.960	− 1.883	0.060
Grade [*n* (%)]			17.594	<0.001
Junior Year 1	324 (36.8)	431 (28.8)		
Junior Year 2	313 (35.5)	564 (37.7)		
Junior Year 3	244 (27.7)	500 (33.4)		
Growing environment [*n* (%)]			10.688	0.001
Urban	81 (9.2)	205 (13.7)		
Rural	800 (90.8)	1290 (86.3)		

### NSSI modality, behavioral purpose, external stimulus event statistics

This survey showed that 67.4% (*n* = 594) of people used multiple means of self-injury. The most common form of NSSI was hair pulling (51.0%), followed by cutting the skin with an object such as a pocket knife (43.0%) and hitting a harder object such as a wall with the hand (42.8%). Among the behavioral purposes of NSSI (Table 2), the most common was to vent bad emotions or feelings (57.5%), followed by relieving feelings of loneliness, numbness or nervousness (29.4%) and punishing oneself for a fault (25.2%). Poor academic performance, reprimand from parents or teachers, and too much pressure from studies or employment were the three most common events that stimulated the occurrence of NSSI (Table 3).

**Table 2:** Purpose of NSSI

Purpose	Number (*n*)	Percentage (%)
Venting a bad mood or feeling	507	57.5
Relieves feelings of loneliness, numbness or nervousness	259	29.4
Punishing yourself for a fault	222	25.2
Determined to do something	163	18.5
Running away from something you don't like	147	16.7
Exercise some kind of ability	145	16.5
Seeking excitement or pleasure	114	12.9
Being understood by others	104	11.8
To spur or encourage yourself	92	10.4
Letting others know how you feel	77	8.7
Get the attention of others	70	7.9
Get satisfaction	67	7.6
Find something fun to do	57	6.5
Express your determination	53	6.0
Control of the situation	51	5.8
Integration into a group	47	5.3
Other	136	15.4

**Table 3:** External factors/events of NSSI

External factors/events	Number (*n*)	Percentage (%)
Poor academic performance	396	44.9
Parent or teacher reprimand	313	35.5
Too much pressure to study or get a job	310	35.2
Poor relationship with others	240	27.2
Poor relationships between family members	174	19.8
Difficulty adapting to new living conditions	83	9.4
Loss of love or separation from a lover	64	7.3
Economic hardship	52	5.9
Other	147	16.7

### Correlation analysis of negative life events, emotional symptoms and NSSI

Pearson's correlation analysis of negative life events, anxiety, depression and NSSI showed a significant positive correlation between negative life events, anxiety, depression and NSSI (*P* < 0.01; Table 4), i.e., the more negative life events experienced and the heavier the impact, the more adolescents tended to develop anxiety and depression and the greater the severity of their NSSI. The correlation coefficients were 0.706 for anxiety and depression, 0.424 for anxiety and NSSI, and 0.437 for depression and NSSI. The correlation coefficients of each dimension of negative life events with anxiety, depression, and NSSI are shown in Table 4.

**Table 4:** Correlation analysis of negative life events, emotional symptoms and NSSI

	$\overline{x}$ ± *s*	Interpersonal relations	Academic stress	Punished	Loss	Other	Total Stress	Anxiety	Depression	NSSI
Interpersonal relationship	9.30 ± 5.420	1								
Academic Stress	9.00 ± 4.986	0.730**	1							
Punished	9.42 ± 7.979	0.736**	0.756**	1						
Loss	3.43 ± 3.647	0.535**	0.539**	0.584**	1					
Other	4.90 ± 4.311	0.662**	0.592**	0.801**	0.458**	1				
Total stress	37.81 ± 23.229	0.862**	0.853**	0.910**	0.730**	0.823**	1			
Anxiety	46.62 ± 9.466	0.384**	0.412**	0.310**	0.164**	0.355**	0.394**	1		
Depression	53.01 ± 10.643	0.389**	0.356**	0.317**	0.152**	0.406**	0.392**	0.706**	1	
NSSI	8.01 ± 13.866	0.293**	0.252**	0.253**	0.146**	0.348**	0.311**	0.424**	0.437**	1

**P < 0.01

### Structural equation modeling analysis

The above results mainly show a direct relationship between negative life events, anxiety, depression and NSSI. To further explore whether there is an indirect relationship between negative life events, anxiety, depression and NSSI, we developed a structural equation model for analysis.

**Fig. 1:**
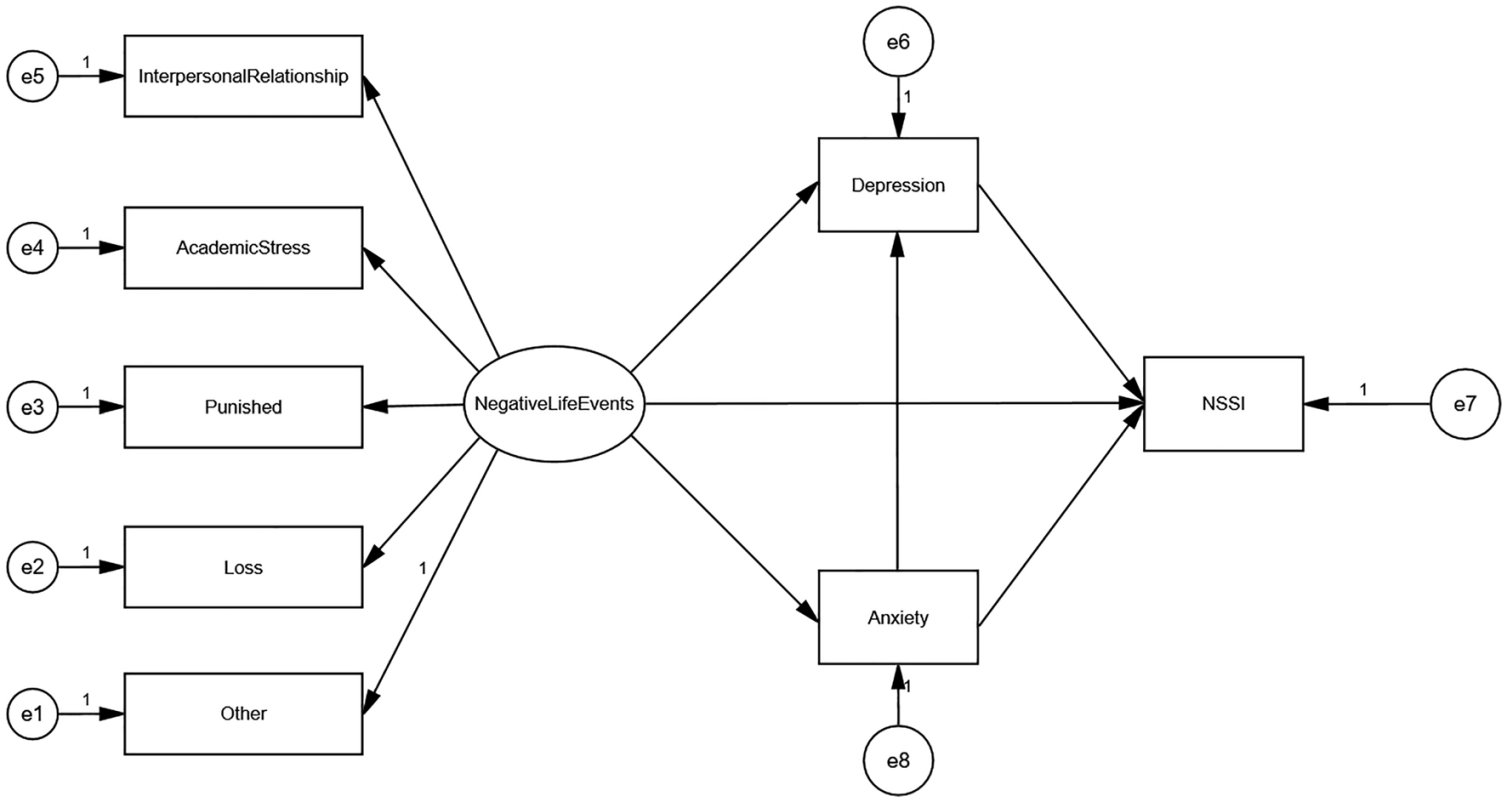
Theoretical assumptions model

Based on the assumptions of previous research theories, we constructed the following theoretical model (Fig. 1) using the five factors of interpersonal relationship, academic stress, punished, loss, and other as the five observed variables of the latent variable negative life events.

The theoretical model includes the following three hypotheses:

- H1: Negative life events, anxiety, and depression have a direct effect on NSSI.
- H2: Negative life events have an indirect effect on the NSSI through anxiety or depression.
- H3: Negative life events can also affect depression through anxiety, which in turn has an indirect effect on the NSSI.

**Fig. 2:**
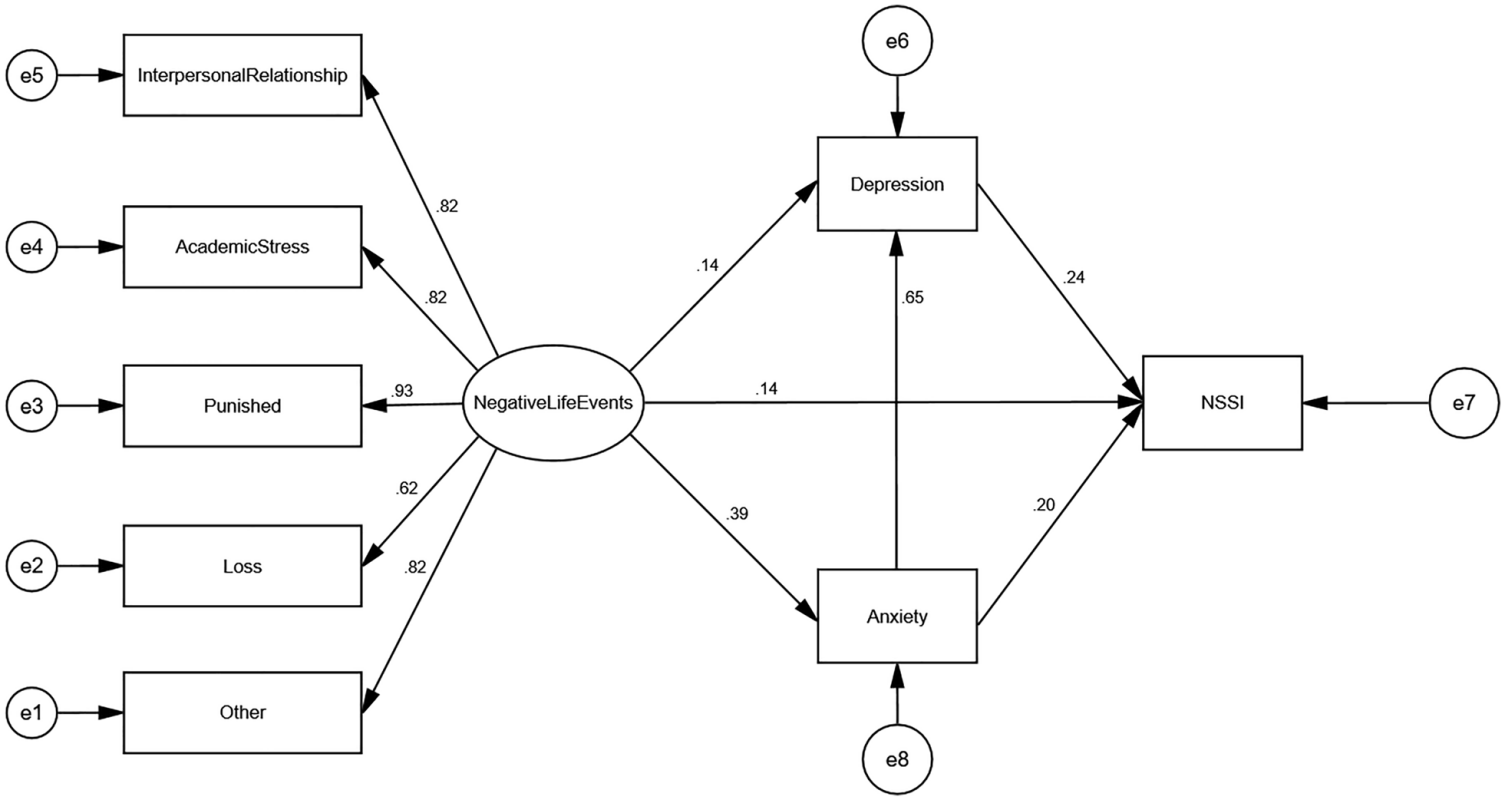
Total sample fitting model before correction

**Fig. 3:**
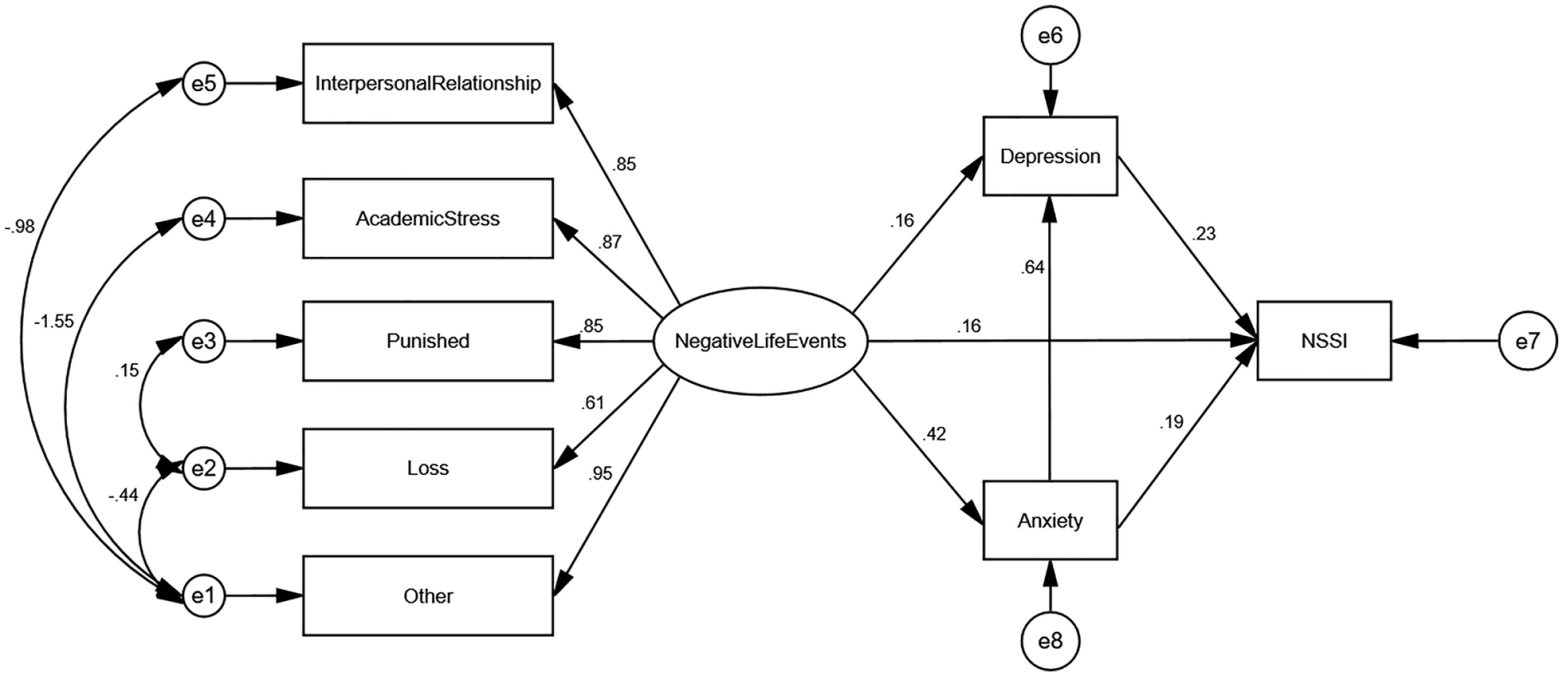
Modified total sample fitting model

Model I (Fig. 2) was obtained by fitting the theoretical hypothesis model of Fig. 1 using maximum likelihood estimation. The results showed that the fit indices of the model were: RMSEA = 0.132, AGFI = 0.849, GFI = 0.928, CFI = 0.935, NFI = 0.931, TLI = 0.893, IFI = 0.935 and SRMR = 0.046, where the four items of GFI, CFI, NFI and IFI met the criteria of >0.9 for model fitness and the model fit was fair. In previous studies, van Oort FV et al. showed that adolescent mental health is closely related to parenting styles [43], unhealthy parenting styles may cause more stress and psychological harm to the adolescent. In a study by Underwood MK and Alexander JD et al. personality traits were correlated with “adolescent maladjustment” and “breakups with partners” [44, 45]. The research of study habits by Yang et al. showed that study habits can affect adolescents' academic performance and make them receive criticism. At the same time, Yang et al. pointed out that study habits are the individual's attitude and method of learning in previous activities, and therefore affect whether adolescents have dropped behaviors in their academic life [46]. Finally, Burger C et al.'s study of school management styles suggests that school management styles may lead to different interpersonal outcomes, which in severe cases may cause students to erupt in conflict and may also produce some poor relationships in social [47]. Based on the above study, we consider that the “personality traits” not included in ASLEC could influence both the interpersonal relationship factor (disputes with classmates) and the other factor (loss of love), so the unexplained parts of these two factors may be related. Similarly, “parenting styles” can affect both the academic stress factor (parental pressure to advance to higher education) and other factors (parental scolding); “Study habits” can affect both the punished factor (losing something) and loss factor (being criticized); “School management styles” can affect both loss factor (be stolen) and the other factor (fighting with others). The residual correlation between the factors was established based on the above. The model was modified to obtain Model II (Fig. 3), which had a fit index of RMSEA = 0.073, AGFI = 0.945, GFI = 0.980, CFI = 0.985, NFI = 0.982, TLI = 0.968, IFI = 0.985 and SRMR = 0.033. The model fit criteria of RMSEA < 0.08, AGFI, GFI, CFI, NFI, TLI, IFI > 0.9, and SRMR < 0.08 were met. The model fits the data well and each of these standard path coefficients reached a significance level of 0.01.

Model II showed that the standard path coefficients for the direct effects of negative life events, anxiety and depression on the NSSI were 0.16, 0.19 and 0.23, respectively, which supported the hypothesis of H1. Negative life events can have an indirect effect on the NSSI through anxiety and depression, respectively, with an effect strength of 0.42 × 0.19 = 0.080 and 0.16 × 0.23 = 0.037, which supports the hypothesis of H2. Negative life events can also affect depression through anxiety, which in turn affects NSSI, with an effect size of 0.42 × 0.64 × 0.23 = 0.062, and the above findings support the hypothesis of H3. Summarizing the above findings, we conclude that there are six pathways for the effects of negative life events, anxiety and depression on NSSI as follows: “Negative life events → NSSI”; “Anxiety → NSSI”; “Depression → NSSI”; “Negative life events → anxiety → NSSI”; “Negative life events → depression → NSSI”; “Negative life events → anxiety → depression → NSSI”. Anxiety and depression play an incomplete mediating role between negative life events and NSSI.

## Discussion

The main objective of this study was to investigate the interrelationship between negative life events, emotional symptoms (anxiety, depression) and NSSI, and the mediating role that emotional symptoms play between negative life events and NSSI. We found that negative life events and emotional symptoms all have a direct effect on NSSI. In addition, negative life events also have an indirect effect on NSSI via emotional symptoms, in which emotional symptoms play an incomplete mediating role.

In the present study, the prevalence of NSSI among Chinese junior high school students was 37.1%. Results from a previous study of rural Chinese adolescents showed a 29% prevalence of NSSI [48]. A study of adolescents in the United States showed that 6.4%-30.8% of adolescents had an NSSI in the past year [25]. In addition, the results of national studies on the prevalence of NSSI fluctuate from 17.1 to 38.6% [49]. The reason why the results of this study were higher than those of previous studies may be due to the differences in the criteria for defining and measuring NSSI. For example, in Tang et al.'s study, the criterion for self-injury was defined as “occurring more than 5 times per year”, whereas in Martin et al.'s study, the instrument used to measure NSSI was a question that asked, “During the past 12 months, how many times did you do something to purposely hurt yourself without wanting to die, such as cutting or burning yourself on purpose?” The prevalence of NSSI is higher in girls than in boys, which is the same as previous studies [50, 51]. Previous research has shown [52] that men are more likely to take direct action, i.e., action to change the problem, in response to life events than women, so that when faced with negative life events, boys are more likely to take action to solve the problem, whereas girls may need more time to deal with these negative life events. In addition, the differences in cognitive styles between men and women [53] may also have a certain degree of influence. In terms of the grade distribution of the subjects, the distribution of the group with NSSI was more skewed towards the lower grades, which is similar to the results of previous studies on Chinese secondary school students [54]. The reasons for this may be due to the fact that some adolescents' self-injurious behavior is only a one-time event, etc. The specific causes of which need to be further studied. The higher prevalence of NSSI among rural adolescents than urban supports the findings of previous studies [48]. Compared to urban families, rural families tend to face greater economic stress and therefore may be exposed to more external stimuli, such as being discriminated against by others, family economic difficulties, family exerted greater pressure on study, etc. Pulling hair, cutting the skin with objects such as knives and hitting harder objects such as walls with hands were the three most common ways of NSSI. Venting negative emotions is the most common purpose of NSSI, which is the same as the most common purpose of NSSI described in the DSM-5. Pulling hair, cutting skin with objects such as knives and hitting harder things such as walls with hands are all relatively easy ways to perform NSSI, so all three are the most common ways to perform NSSI. What we must recognize, however, is that any of the self-injury modalities, no matter how much or how little damage is caused, should be taken seriously by us. Any type of self-injury is a sign that the child is indeed self-injuring and sends the message that “this child is currently in distress” and needs our help. Poor academic performance, reprimands from parents or teachers, and too much academic or employment pressure were the most common external factors/events, which were related to our subjects' status as students. Middle school students are in the transition period between primary and high school, and parents often have high expectations for their children. A previous study of Chinese students showed that study stress is a risk factor for emotional problems [55]. Junior high school students face a sudden increase in academic pressure, which inevitably leads to fluctuations in academic performance and the psychological state, as well as possible reprimands from parents and teachers, so the three most common external stimuli are all related to studies.

In this study, negative life events include the interpersonal relationship factor, the academic stress factor, the punished factor, the loss factor and the other factors. For adolescents, interpersonal relationships are mainly reflected in getting along with peers at school, which is an important part of their social life. Learning plays an important role in the lives of adolescents. Junior high school students face increased pressure to study as well as the pressure of entrance exams and are vulnerable to punishment from their parents when their academic performance fluctuates. The loss factor is mainly reflected in the illness or death of a relative or friend, or the loss of something, all of which have a negative and pronounced effect on the adolescent. In particular, the death of a family member or friend can be a very serious psychological shock for adolescents who are exposed to such events for the first time. Other factors include dislike of school, unhappy relationships, fighting with others, and being scolded by parents, which are also common academic, emotional, social and family problems for adolescents.

The present study showed that negative life events, anxiety, and depression were all direct risk factors for NSSI, which validated our H1 hypothesis. The greater the number of negative life events, the greater the degree of NSSI, which is similar to previous studies. A previous study of Chinese children has shown that individuals who have experienced more negative life events are more likely to develop NSSI [56]. Emotional symptoms, mainly anxiety and depression, are widespread among adolescents [57–59]. A previous study of Australian students showed that the detection rate of anxiety co-morbid depression was 33.2% [60]. In addition, the findings for American students also demonstrated that the detection rate of anxiety co-morbid depression was higher [61], and anxiety and depression were risk factors for the occurrence of NSSI [62], and adolescents with more emotional symptoms also tend to have a higher prevalence of NSSI, which is similar to the results of this study.

In this study, negative life events and emotional symptoms can all have a direct impact on NSSI. At the same time, emotional symptoms play an incomplete mediating role in negative life events and NSSI. Previous studies have shown that when adolescents encounter negative life events, they can produce emotional symptoms such as anxiety and depression [63, 64], which in turn are closely associated with NSSI [65, 66]. This validates our H2 hypothesis that negative life events can have an effect on NSSI through anxiety or depression. In addition, Koen et al. mentioned that anxiety and depression can occur together, and in patients with co-morbid anxiety and depression, anxiety often precedes depression [67]. This also validates our H3 view that negative life events, when acting on emotional symptoms, can have an impact on depression through anxiety and thus on NSSI.

Negative events, no matter how big or small, are inevitable in the daily life of young people. As parents or teachers, they should reduce the occurrence of controllable negative life events as much as possible in their lives, such as avoiding physical punishment when children make mistakes and adopting rational education; when children encounter problems in school, parents should communicate with teachers in a timely manner and coordinate with each other to solve problems, etc. This can reduce the frequency of negative life events and thus reduce the impact of negative life events on NSSI. When a negative life event occurs, timely understanding of the child's psychological state and effective communication can effectively reduce the impact of the event on the child's psychological state and prevent the development of anxiety and depression. At the same time, it is important to develop resilience in the face of adversity in children's lives, as previous research [18] has shown that enhancing “resilience” can effectively buffer the link between negative life events and adverse outcomes in adolescents. The most effective way to address anxiety and depression in adolescents is to bring them to a medical professional in a timely manner, both to reduce the impact of negative life events on NSSI in a mediated way and to reduce the impact of emotional symptoms on NSSI directly.

This study has the strength of using a large sample of Chinese secondary school students and using structural equation modeling to explore the mediating role of emotional problems in the relationship between negative life events and NSSI. However, this study also has some limitations. First, the three schools participating in the study were chosen for convenience; second, this survey is a retrospective self-assessment survey, and there may be recall bias as well as the possibility of masking; last, the proportion of urban and rural samples included in this study is not balanced. In future studies, the sample size can be further expanded, the proportion of urban and rural children can be balanced, more longitudinal surveys can be conducted for comparison, and objective indicators such as relevant blood samples, genetic tests, and brain function scans can be improved to find changes in specific blood indicators, Genetic alterations or alterations in specific brain regions for targeted diagnosis and treatment.

In summary, NSSI in Chinese adolescents is still a behavioral problem that must be taken seriously and has a higher prevalence among girls and rural adolescents. Negative life events and emotional symptoms are all direct risk factors for NSSI, and emotional symptoms play a mediating role in the relationship between the effects of negative life events and NSSI. This indicates that for improving NSSI caused by negative life events, the desired outcome can be achieved by intervening on both negative life events and emotional symptoms. Therefore, in the developmental education of adolescents, it is important to both minimize the generation of negative life events, such as improving school rules and regulations to avoid school bullying; avoiding physical punishment to educate children, etc., and to pay attention to the emotional symptoms of children. A healthy child is not only physically healthy, but also psychologically healthy. As Bartoli et al. mentioned, good psychosocial intervention and suicide prevention should be carried out during adolescents' development [68], which reminds us that when serious emotional or behavioral problems arise, guardians should promptly take them to professional medical institutions for diagnosis and treatment to avoid self-injury or suicidal behavior. Early intervention for emotional and behavioral symptoms can also effectively promote the recovery of adolescents and help them return to a normal social life at an early stage.

## Data Availability

The data that support the findings of this study are openly available on request from the corresponding author.
